# The Largest Tubal Pregnancy: 14th Week

**DOI:** 10.1155/2020/4728730

**Published:** 2020-05-20

**Authors:** Amr Elmoheen, Waleed Salem, Mahmoud Eltawagny, Rehab Elmoheen, Khalid Bashir

**Affiliations:** Hamad Medical Corporation, Qatar

## Abstract

Subsequent development and implantation of embryo outside the uterine lining are defined as an ectopic pregnancy. Ectopic pregnancies have a wide range of presentations, for example, acute hemoperitoneum to chronic ectopic pregnancy. The case presented is an unusual case of ectopic pregnancy with large hematosalpinx with classic symptoms. To the best of the authors' knowledge, this case is the largest intact tubal ectopic pregnancy reported ever in the 14^th^ week of gestation. A 40-year-old patient presented to the emergency department with lower abdominal pain, mild dysuria, and loose motion. The patient's previous menstrual cycles were regular till four months ago, then started to be irregular, and she had no history of chronic diseases except repeated pelvic inflammatory diseases (PID). Clinically, the patient was hemodynamically stable. On palpation, the abdomen was tender, and cervical movements were not tender. BHCG in the blood came very high. The bedside point-of-care ultrasound (POCUS) showed free fluid in the abdomen and a sac in the left adnexa with a living fetus (visible heartbeats). The conventional ultrasound showed 14 weeks of an extrauterine gestational sac with visible early pregnancy. Differential diagnosis was either an abdominal pregnancy versus a complicated tubal pregnancy. The surgical pathology report confirmed the diagnosis of ectopic tubal pregnancy as the tube was dilated in the middle portion containing chorionic villi, decidual reaction, and the whole gestational sac consistent with the ectopic tubal pregnancy. The patient had a successful laparotomy with salpingectomy and hemostasis and did well after the operation. So, an intact ectopic tubal pregnancy may last until the 14^th^ week of gestation.

## 1. Introduction

Ectopic pregnancy, according to the Centers for Disease Control and Prevention, accounts for 2% of all reported pregnancies in the United States, approximately [1]. The most common site of the ectopic pregnancy is the fallopian tubes. The other places of implantation include the ovary, cervix, cesarean-section scar, and the abdomen [2]. Commonly, the patient presents with abdominal pain, amenorrhea, and sometimes vaginal bleeding. The patient may have an atypical presentation or even be asymptomatic in the earlier stages [2].

The recent advances of the diagnostic modalities helped in the early diagnosis of the ectopic pregnancy. To the best of the authors' knowledge, this case is the largest intact tubal ectopic pregnancy reported ever in the 14^th^ week of gestation.

## 2. Patient Information

A 40-year-old patient presented into the emergency department with lower abdominal pain, mild dysuria, and loose motion. She had no significant medical or obstetric history except for repeated attacks of pelvic inflammatory diseases (PID) and did not use contraceptives. The patient reported her menstrual cycles as regular until four months ago, then started to be irregular. The patient described the lower abdominal pain as cramping and denied any previous symptoms.

## 3. Clinical Finding

The vital signs of the patient in the emergency department were stable. Her blood pressure was 112/75 mmHg, pulse was 99 beats per minute, respiratory rate was 18 per minute, and there was no fever. She was anxious and in mild pain. She was fully conscious. Her chest was clear, with no associated sounds. Her abdomen was not distended with diffuse tenderness, especially in the umbilical region. She was guarding with no rigidity.

Bedside point-of-care ultrasound (POCUS) showed free fluid in the pouch of Douglas and a sac in the left adnexa with a living fetus and visible heartbeats.

## 4. Diagnostic Assessment and Investigations

The lab results were positive for anemia. The beta-human chorionic gonadotropin (BHCG) level was 56748.0 mIU/ml. The other blood investigations were within normal.

**Figure 1:**
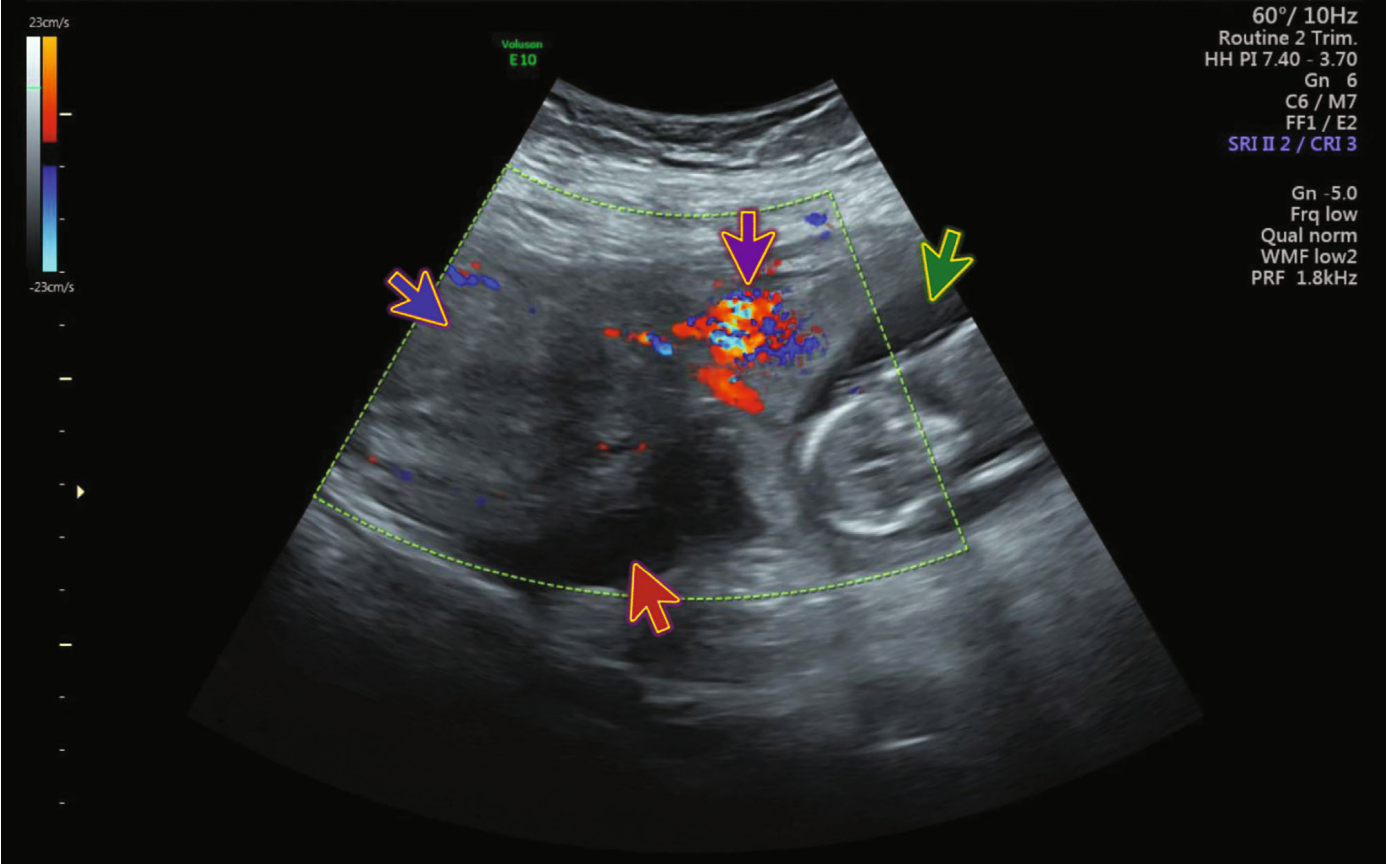
Ultrasound shows the gestational sac (green arrow) that was seen towards the left side of the uterus connected through a vascular stalk (purple arrow) to the uterus (blue arrow) and free fluid in the pelvis (red arrow).

**Figure 2:**
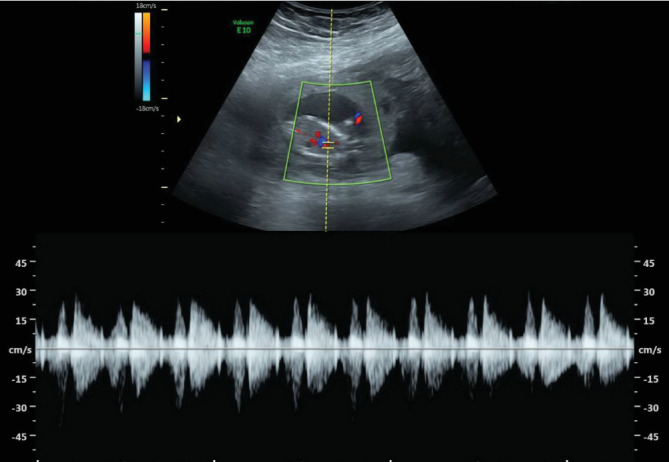
Ultrasound shows extrauterine pregnancy and the fetal heart rate (176 bpm).

**Figure 3:**
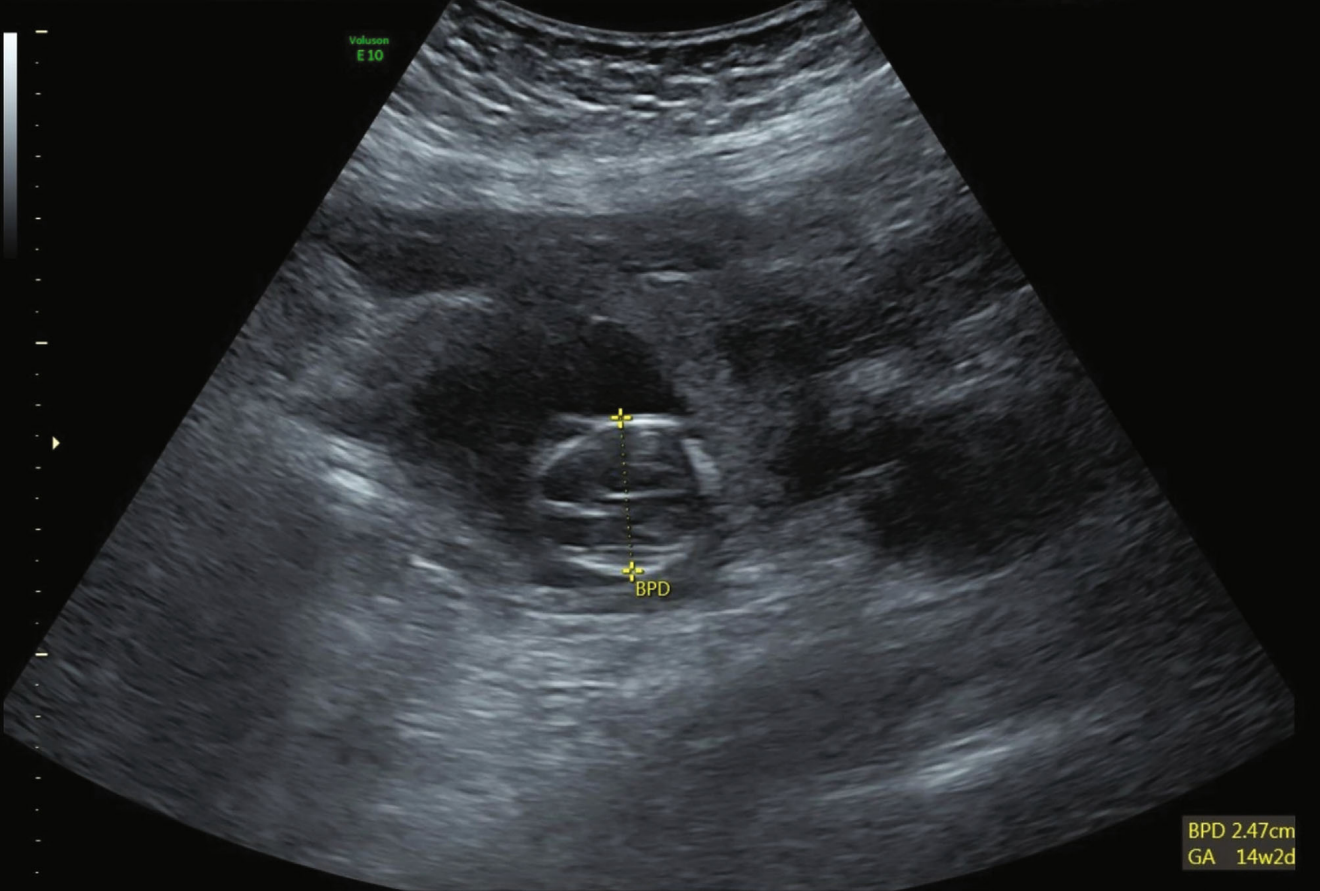
Ultrasound shows the biparietal diameter (BPD) measuring 2.47 cm which equals 14 weeks and 2 days.

**Figure 4:**
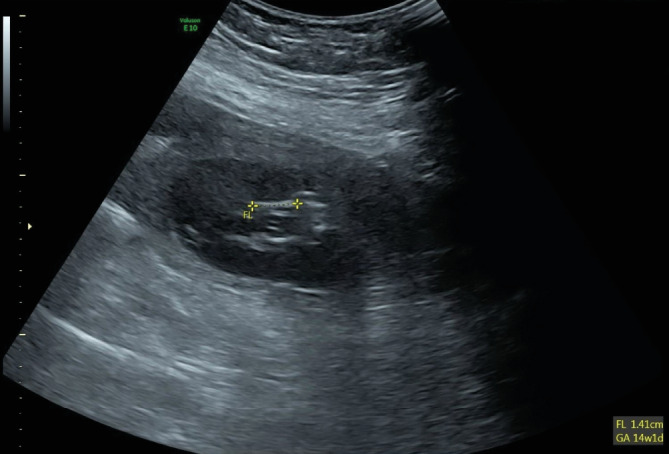
Ultrasound shows the femur length (FL) measuring 1.41 cm which equals 14 weeks and 1 day.

Ultrasound of the abdomen and pelvis showed evidence of an extrauterine gestational sac hosting a viable fetus of 14-week and 1-day gestation. The gestational sac was seen towards the left side of the uterus connected through a vascular stalk to the uterus, as shown in (Figure 1). The fetal cardiac pulsations were recognized and recorded. The fetal heart rate was 176 bpm (Figure 2). The biparietal diameter (BPD) measured 2.47 cm, which corresponded to 14 weeks and 2 days (Figure 3). The femur length (FL) measured 1.41 cm, which corresponded to 14 weeks and 1 day (Figure 4). Moderate hemoperitoneum in the pouch of Douglas was noted and could represent oozing or rupture of the gestational sac. There were no blob sign or bagel sign. There were no fluids in the Morison pouch. The anteverted uterus measured 8.1 × 6.6 cm and showed an endometrial thickness of 12.7 mm and an empty uterine cavity.

The surgical pathology report showed that the specimen consists of a possible female fetus with an attached umbilical cord and detached fragmented placenta. The fetus weighed 26.5 grams and measured 7.2 cm crown to rump and 3.7 cm rump to heel. The foot measured 1.1 cm in length. The head circumference measured 8.5 cm. There was no evidence of cleft palate grossly identified. The vertebral column was intact. The fetal anus and mouth were patent. The skin was focally hemorrhagic. There were four extremities present, each bearing five digits. There was evidence of a possible female genitalia present. The trimmed placenta weighed 73 g and measured 9.5 × 5 × 2.2 cm. The umbilical cord measured 3.6 cm in length and 0.4 cm in diameter and was normally coiled. The left fallopian tube measured 11.5 cm in length and 10 cm in diameter and was dilated in the middle portion containing chorionic villi, decidual reaction, and the whole gestational sac consistent with ectopic tubal pregnancy.

## 5. Therapeutic Intervention

In the presence of anemia, the patient received blood products. The patient had a laparotomy exploration for fearing of any unexpected bleeding because of the advanced pregnancy. A salpingectomy with hemostasis was done successfully.

## 6. Follow-Up and Outcome

During the postoperative period, the patient recovered and did not suffer any complications. She was discharged home on the 4th day postoperatively.

## 7. Discussion

Up to 2% of gestations could be an ectopic pregnancy [1]. The prevalence of ectopic pregnancy is as high as 18%, with women presenting to the emergency department with abdominal pain and vaginal bleeding in the first trimester or both [3]. Despite the improvements in diagnosis and treatment, ruptured ectopic pregnancy continues to have significant morbidity and mortality. Ruptured ectopic pregnancies, from 2011 to 2013, accounted for 2.7% of all pregnancy-related deaths and, moreover, are the leading cause of mortality related to hemorrhage [4].

There are no known risk factors in half of all the women who receive a diagnosis of ectopic pregnancy. Women who had a previous ectopic pregnancy are at an increased risk of recurrence. There is a chance of approximately 10% of recurrence in women with a history of ectopic pregnancy. Besides, the risk of recurrence increases to more than 25%. Previous pelvic or fallopian tube surgery, factors secondary to ascending pelvic infection, and prior damage to fallopian tubes are important risk factors for ectopic pregnancy [5]. Certain factors in women who become pregnant through the use of assisted reproductive technology, for example, multiple-embryo transfer and tubal factor infertility, are associated with an increased risk of ectopic pregnancy [6]. Independent of how they become pregnant, women with a history of infertility are also at an increased risk of ectopic pregnancy. Similarly, other less significant risk factors of ectopic pregnancy are age older than 35 years and cigarette smoking. Women using an intrauterine device (IUD) compared to women not using any form of contraception are at a lower risk of ectopic pregnancy; IUDs are highly effective for the prevention of pregnancies. However, 53 percent of the pregnancies which occur in the presence of an IUD are ectopic [7]. Other factors, for example, cesarean delivery, pregnancy loss, previous elective pregnancy termination, emergency contraception failure, and oral contraceptive use, are not associated with ectopic pregnancy [8]. An ectopic pregnancy can also occur with an intrauterine pregnancy; the condition is known as heterotopic pregnancy. Among women with a naturally achieved pregnancy, the risk of heterotopic pregnancy is estimated to range from 1 in 4,000 to 1 in 30,000. Moreover, the 1 in 100 women who have undergone in vitro fertilization is at risk of ectopic pregnancy [9].

The fallopian tube is the most common location of ectopic pregnancy and accounts for more than 90% of the cases. Previous cesarean scar, ovarian, cervical, and abdominal implantations have also been reported [2, 10–12]. Moreover, because of delayed diagnosis and treatment, ectopic pregnancy often results in more significant morbidity, with 1% case of implantation in the abdomen, 1% in the cervix, and 1-3% in the cesarean scar [13]. A hinged or disrupted fallopian tube anatomy is a risk factor predisposing women to ectopic pregnancies. Sexually transmitted infections, prior miscarriages, elective abortions, and previous ectopic tubal pregnancies are also influencing factors of ectopic pregnancies [11, 14].

Furthermore, presenting symptoms and reported risk factors used for the monitoring of ectopic pregnancies have limitations of their own. The patients are previously asymptomatic; similarly, ruptured ectopic pregnancies are positive in the case of advanced maternal age. Also, it is equally important to closely monitor individuals who are at low risk of ectopic pregnancy. Patients with ruptured ectopic pregnancies have had either a previous ectopic pregnancy or adhesion on the same side or have abnormal anatomy of the reproductive system; this increases the likeliness of ectopic implantation.

Like diagnosis, the management of ectopic pregnancy has been improved with the advances in medicine; however, despite it, it still carries its risks. In cases where the gestation appears as an extrauterine lesion in a woman who is stable hemodynamically, it measures smaller than 3 cm. The patients are amenable to medical or noninvasive management. However, if the patient is unstable hemodynamically and the extrauterine lesion is larger than 3 and 1/2 cm, it is recommended to approach the case with surgical management. The preferred surgical approaches include salpingotomy or salpingectomy, a preferred surgical option in women who are stable hemodynamically; moreover, these options are less invasive than open surgery. If the bleeding, however, is uncontrolled and the ectopic pregnancy cannot be excised adequately, the case can warrant open surgery [15]. Expectant treatment and administration of methotrexate either systematically or injection locally in the gestational sac itself are alternative therapies to ectopic pregnancy [16, 17].

Cases of large ectopic pregnancies, other than the fallopian tube, have been previously published, which are accommodating and more distensible for a developing fetus [18–20]. However, there is limited literature on large tubal ectopic pregnancies.

POCUS is a crucial tool for every emergency physician as it can decrease the time to diagnosis and to direct patient care, especially in life-threatening conditions. It can detect the rupture of the ectopic pregnancy and the ongoing intra-abdominal bleeding, which can be a sign of operative intervention. To diagnose ruptured ectopic pregnancy, POCUS has high sensitivity to detect an empty uterus, free fluid, extrauterine mass, or gestational sac of the ectopic pregnancy [21]. Because of the large size of the tubal pregnancy, the transabdominal ultrasound could not precisely differentiate between abdominal and tubal pregnancy. Transvaginal ultrasound examination and measurement of the increasing level of the *β*-subunit of human chorionic gonadotropin have more sensitivity and specificity than the transabdominal ultrasound in the diagnosis of ectopic pregnancy [22].

In 2015, a report was published, which describes a nonruptured ectopic pregnancy with twins; the fetal crown-rump length was 2 cm, which means 8 weeks and 4 days of gestation [23]. Another study reported an unruptured gestational sac of a bilateral tubal ectopic pregnancy with sacs over 4 cm and proved to be 7 weeks and 6 days of gestation [24].

There are not many publications detailing a tubal pregnancy of over 10 weeks, as seen in this case report. In addition, studies describing symptoms of ectopic pregnancies are also sparse. While the techniques of diagnosis have improved over time, cases such as these can go unnoticed, or the diagnosis is not without error. Furthermore, ultrasound is not able to detect intrauterine pregnancies, necessarily, especially below the beta-HCG discriminatory zone.

In 2016, a 36-year-old woman presented with lower abdominal pain and vaginal bleeding, and the ultrasound showed a fetal crown-rump length (CRL) of 2.02 cm, corresponding to 8 weeks and 4 days. She has left laparoscopic salpingectomy and successfully recovered [25].

In 2018, a 35-year-old woman presented with sudden suprapubic abdominal pain, vomiting, and vaginal spotting, while the ultrasound showed a left adnexal ectopic pregnancy with a crown-rump length which was over 41 mm, corresponding to 11 weeks. Exploratory laparotomy was done. She was successfully discharged home on the 4th day postoperatively [26].

In 2019, the previous largest ruptured tubal pregnancy was reported by Kim and his team. A 39-year-old woman presented with several fainting attacks, abdominal pain, and vaginal bleeding. Her beta-human chorionic gonadotropin (BHCG) level was 55713 mIU/ml. Ultrasound showed a right adnexal mass. The biparietal diameter (BPD) of the fetus measured 2.2 cm, corresponding to 13 weeks of gestation. Emergency laparoscopic surgery was performed [27].

To the best of the authors' knowledge, our case is the largest tubal ectopic pregnancy reported ever in the 14th week of gestation. The patient's beta-human chorionic gonadotropin (BHCG) level was 56748.0 mIU/ml. The biparietal diameter (BPD) of the fetus measured 2.47 cm, corresponding to 14 weeks and 2 days. The femur length (FL) measured 1.41 cm, corresponding to 14 weeks and 1 day. The surgical pathology report confirmed the ectopic tubal pregnancy as the fallopian tube contained chorionic villi, decidual reaction, and gestational sac consistent with the ectopic pregnancy.

## 8. Conclusion/Take-Home Messages

Rupture ectopic pregnancy can cause intraperitoneal bleeding and hemorrhagic shock. Early detection of ectopic pregnancy is essential for the reduction of maternal morbidity and mortality. Bedside point-of-care ultrasound (POCUS) has a crucial role in the diagnosis of ectopic pregnancy. Intact tubal ectopic pregnancy may last till the 14^th^ week of gestation.

## 9. Patient Perspective

The patient said, “Being 40 years old and having children in their adulthood time, I am surprised that I am even pregnant. I did not focus on the change in the amount of my menstruation period. I am worried about the possibility of bleeding during the operation.” After the operation, she said, “I am very thankful to the medical staff for their great efforts they did to save my life.”
